# Barriers to commercial deployment of biorefineries: A multi-faceted review of obstacles across the innovation chain

**DOI:** 10.1016/j.heliyon.2024.e32649

**Published:** 2024-06-07

**Authors:** Denzel Christopher Makepa, Chido Hermes Chihobo

**Affiliations:** Department of Fuels and Energy Engineering, Chinhoyi University of Technology, Private Bag, 7724, Chinhoyi, Zimbabwe

**Keywords:** Biorefinery, Sustainability transitions, Technology commercialization, Bioeconomy, Bioenergy, Innovation barriers

## Abstract

Realizing integrated biorefineries producing multiple fuels, chemicals and materials from sustainable biomass feedstocks holds promise for transitioning industries onto low-carbon trajectories. However, widespread commercial implementation remains elusive despite two decades of technological advancements. This review synthesizes current literature to provide a comprehensive analysis of key multi-dimensional barriers inhibiting the scale-up of biorefineries. The review discusses the technical challenges around biomass conversion processes. Economic viability concerns such as high capital costs and lack of market competitiveness are also assessed. The review also evaluates the regulatory and policy complexities that poses risks and uncertainties in the scaling up of biorefineries. Socio-political acceptance hurdles including community engagement and public perception are also reviewed. The interconnected nature of these challenges is emphasized and strategies are recommended to enable full potential realization, covering areas such as enhanced stakeholder collaboration, advanced process intensification, supportive policy frameworks, innovative financing models and strategic marketing initiatives. International pilots and cross-sectoral knowledge exchange are highlighted as priority enablers. In conclusion, this review synthesizes insights from extensive demonstration efforts to identify priorities and pathways for accelerating the global commercial transition towards sustainable biorefinery implementation. It aims to inform strategic decision-making and collaborative actions amongst stakeholders in research, industry and policy domains.

## 1 Introduction

The last few decades have seen a notable rise in global consciousness around the need to transition away from non-renewable, carbon-intensive economies towards more sustainable systems capable of meeting social and economic needs within planetary boundaries [1]. Spiraling environmental crises such as climate change, biodiversity loss and resource depletion have underscored the urgency of transitioning our production and consumption patterns.

At the same time, growing populations and rising living standards worldwide are intensifying pressures on natural capital and ecosystems [2]. It is projected that global resource extraction will increase over 90 % by 2060 if business-as-usual continues, severely exacerbating environmental degradation if left unchecked. However, international commitments like the Paris Agreement and UN Sustainable Development Goals indicate a shared global vision for decarbonization, protection of nature and establishment of regenerative economies [3].

The bioeconomy has been highlighted as a key strategic framework by many governments and intergovernmental bodies, with the aim to address environmental, social, and economic needs [4]. A robust bioeconomy offers opportunities to steer industries toward sustainability, tackle climate change effects, and improve livelihoods by utilizing renewable biological resources and processes [5]. Transitioning from fossil-based materials and feedstocks to bio-based alternatives can environmentally optimize entire value chains across sectors, resulting in a progressive dematerialization away from carbon-intensive pathways [6].

In the context of urgent global environmental challenges such as climate change, biodiversity loss, and resource depletion, there is a pressing need to transition towards sustainable systems that can meet societal needs within ecological limits [1,2]. This imperative is underscored by projections indicating a substantial increase in resource extraction by 2060 if current trends persist, exacerbating environmental degradation. International agreements like the Paris Agreement and UN Sustainable Development Goals demonstrate a shared commitment to decarbonization and the establishment of regenerative economies. The emergence of the bioeconomy as a strategic framework aligns with these goals, offering opportunities to shift industries towards sustainability, mitigate climate impacts, and improve livelihoods. Biorefineries, as integral components of the bioeconomy, play a crucial role in this transition by converting renewable biomass into value-added products, reducing reliance on fossil resources, and promoting circularity [4]. Despite technological advancements, barriers persist in scaling up biorefineries commercially, encompassing technical complexities, economic viability concerns, regulatory challenges, and socio-political acceptance issues. Addressing these barriers is paramount to realizing the full potential of biorefineries and advancing sustainable industrialization.

As large-scale, integrated hubs for converting sustainable biomass into multiple value-added products, biorefineries are envisaged as core industrial components of the bioeconomy [6]. Moving beyond single-output facilities or combustion models, the concept of advanced biorefineries aims to fully leverage biomass resources through conversion into an array of biofuels, chemicals, materials and other outputs using biological and thermochemical processes.

When supported by sustainable feedstock sourcing and waste recycling schemes, biorefineries offer potential environmental benefits like greenhouse gas mitigation, reduced water usage and improved nutrient management compared to fossil-intensive production [7]. Their establishment can also stimulate rural development and diversification through creation of bioeconomy value chains. By providing renewable alternatives to fossil-derived products, biorefineries aim to decouple industrialization from unsustainable resource extraction while realizing a circular bioeconomy vision [8].

Over the past two decades, considerable research efforts have advanced technological capabilities for lignocellulose fractionation, catalytic conversion processes and other key biorefinery unit operations. Establishment of pilot and demonstration facilities has helped validate process pathways at larger scales and enabled learning. However, widespread commercial realization of advanced integrated biorefineries producing multiple fuels and chemicals from sustainable biomass feedstocks remains elusive to date [9]. While some examples exist employing single conversion pathways to produce commodities like ethanol, optimal technological configurations enabling flexible multi-product integration at industrial scales have yet to be fully attained [10].

Key technical barriers around process intensification, integration and optimization for diverse product slates continue to inhibit successful scale-up [11]. In addition, biorefinery projects face interconnected economic, policy and socio-political uncertainties retarding commercial investments and adoption [12]. Overcoming these challenges constitutes an important focus of ongoing research and demonstration efforts worldwide.

This review presents a novel and comprehensive analysis of the challenges hindering the widespread commercialization of integrated biorefineries. Despite significant technological progress over the past two decades, the commercial implementation of biorefineries capable of producing multiple fuels, chemicals, and materials from sustainable biomass feedstocks remains limited. The review explores the intricate technical challenges associated with biomass conversion processes, integration complexities, and the need to achieve flexible multi-product portfolios at commercial scales. Additionally, it addresses economic viability concerns such as high capital costs and market competitiveness issues. Furthermore, the manuscript evaluates regulatory and policy complexities, as well as socio-political acceptance hurdles including community engagement and public perception. This review not only outlines the challenges but also proposes strategic solutions by synthesizing current literature and highlighting the interconnected nature of these barriers.

## 2 Biorefinery

Biomass is a renewable source of carbon that offers numerous environmental and economic benefits. These include carbon sequestration, production of bioenergy, biofuels, and bioproducts to partially substitute fossil-based products [13]. While biomass holds potential as an energy source, its utilization faces certain limitations. These include the seasonality and geographic variability of supply for many feedstocks, relatively low energy density compared to fossil fuels, as well as constraints associated with large-scale cultivation of feedstock on agricultural lands [14].

The biorefinery concept has increasingly been adopted as it aims to optimize the sustainable utilization of biomass resources. By facilitating the integrated processing of feedstock inputs through diverse conversion methods, biorefineries seek to generate multiple high-value commodity and energy outputs from a given biomass source. This multi-product platform approach allows for a more complete and circular bioeconomy where biomass carbon and nutrients are efficiently reallocated from waste streams into new product pipelines. As such, the biorefinery framework has gained significant attention from researchers and industry players striving for environmental, economic and social sustainability in biomass conversion operations. A biorefinery is a processing facility that takes biomass as feedstock and integrates various conversion technologies to efficiently produce bioenergy, biofuels, and biobased products [15]. Key conversion platforms typically incorporated include thermochemical processes (e.g. combustion, gasification, pyrolysis), biochemical processes (e.g. anaerobic digestion, fermentation), and growth of microorganisms [16].

The evolution of biofuel resources spans four generations, each representing progressive advancements in bioenergy production. Initially, first-generation biofuels were derived from food crops but faced sustainability challenges due to food-versus-fuel competition and land-use changes. Second-generation biofuels shifted focus to non-food biomass sources, aiming for sustainability by utilizing materials like agricultural residues and energy crops. Third-generation biofuels emerged from algae and microorganisms, offering high productivity and reduced pressure on agricultural resources. The latest generation, fourth-generation biofuels, employs advanced processes like synthetic biology and bioconversion technologies, striving for higher energy efficiency and lower environmental impacts, thus marking a significant stride towards sustainable bioenergy solutions [17].

**Fig. 1:**
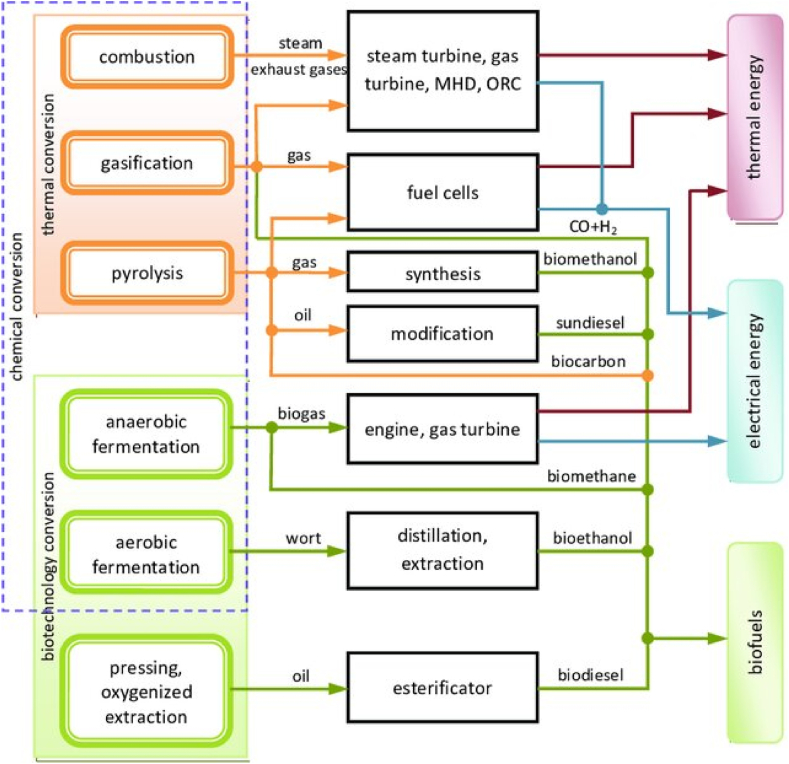
Process flow diagram of a generic integrated biorefinery [18].

Fig. 1 presents a process flow diagram of a generic integrated biorefinery showing the main conversion technologies and product outputs. The modular, flexible design allows processing of different biomass inputs. Examples of feedstocks utilized in biorefineries include lignocellulosic biomass, algal biomass, waste biomass streams and agricultural/forestry residues. Lignocellulosic feedstocks undergo size reduction, pretreatment and fractionation prior to conversion [17]. Algal cultivation and biomass harvesting are integrated steps. Wastes from conversion processes can be recycled back into the system to further improve efficiencies and output yields.

Commercial operations have demonstrated production of biofuels (biodiesel, bioethanol, biogas), power, and a variety of biobased chemicals and materials from platform building blocks. However, large-scale deployment of integrated biorefineries faces technological, economic and other barriers that this review seeks to analyze. The concept represents a shift towards a more circular bioeconomy that sustainably utilizes biomass resources.

### 2.1 Biomass combustion

Biomass combustion is a crucial process in energy production, involving the thermal decomposition of organic materials in the presence of oxygen. The chemical reactions during biomass combustion primarily involve the oxidation of carbon, hydrogen, and other organic compounds present in the biomass. These reactions release heat energy, which can be harnessed for various applications, including electricity generation and heat production [19]. The combustion of biomass can be represented by the simplified chemical equation in Equation (1).

(1) $$Biomass+O_{2}\to {CO}_{2}+H_{2}O+heat$$

In this equation, biomass reacts with oxygen (O_2_) to produce carbon dioxide (CO_2_), water (H_2_O), and heat. However, biomass is a complex mixture of organic compounds, including cellulose, hemicellulose, lignin, and other volatile components. Therefore, the actual combustion process involves a series of more complex reactions, including the combustion of carbon to carbon dioxide and the combustion of hydrogen to water vapor. The reaction kinetics of biomass combustion are influenced by several factors, including temperature, oxygen concentration, and the composition of the biomass [19]. Higher temperatures generally promote faster combustion rates, leading to increased heat release. The presence of sufficient oxygen is also crucial for complete combustion, as inadequate oxygen can result in the formation of carbon monoxide (CO) and other incomplete combustion products.

The percentage conversion of biomass during combustion depends on factors such as the type of biomass, combustion conditions, and the efficiency of the combustion system [20]. Advanced combustion technologies, such as fluidized bed combustion and gasification, aim to improve biomass conversion efficiency and reduce emissions of pollutants such as particulate matter and nitrogen oxides (NOx).

### 2.2 Biomass gasification

Biomass gasification is a thermochemical process that converts biomass into a gaseous mixture called syngas (synthesis gas), consisting mainly of hydrogen (H_2_), carbon monoxide (CO), carbon dioxide (CO_2_), and trace amounts of methane (CH_4_) and other gases [21]. This process occurs under controlled conditions of temperature, pressure, and oxygen availability, typically in the absence of complete combustion.

The chemical reactions involved in biomass gasification can be complex and depend on the specific conditions of the gasification reactor. However, a simplified representation of the gasification reactions is as follows:

***Pyrolysis.*** Biomass is heated in an oxygen-limited environment, leading to the decomposition of organic compounds into volatile gases, tar, and char [22].

(2) Biomass→Volatilegases+Tar+Char

***Gasification.*** Volatile gases and char react with a controlled amount of oxygen or steam to produce syngas through several chemical reactions (Equations (3), (4), (5))) [23].

(3) $$C+H_{2}O\to CO+H_{2}$$

(4) $$C+{CO}_{2}\to 2CO$$

(5) $$C+{CO}_{2}\to {CO}_{2}$$

The reaction kinetics of biomass gasification depend on factors such as temperature, pressure, residence time, and the composition of the biomass feedstock [23]. Higher temperatures and longer residence times generally favor the production of syngas with higher hydrogen content. The percentage conversion of biomass in gasification processes can vary widely depending on the technology used and the operating conditions. Advanced gasification systems, such as fluidized bed gasifiers and downdraft gasifiers, can achieve high conversion efficiencies and produce syngas suitable for a range of applications, including electricity generation, heat production, and synthesis of biofuels and chemicals [24].

### 2.3 Biomass pyrolysis

Biomass pyrolysis is a thermal decomposition process that occurs in the absence of oxygen or with limited oxygen supply, resulting in the production of biochar, bio-oil, and syngas [25]. This process involves heating biomass at high temperatures, typically between 300 °C and 800 °C, leading to the breakdown of complex organic compounds into simpler molecules. The chemical reactions involved in biomass pyrolysis can be categorized into the following main stages [25]:

i. *Drying and preheating.* Initially, moisture present in the biomass is evaporated as the temperature rises, followed by preheating of the biomass.
ii. *Pyrolysis.* Once the biomass is sufficiently heated, it undergoes pyrolysis, where thermal decomposition occurs, and various products are formed. The products formed include solid char/biochar which is a carbon-rich residue remaining after pyrolysis, liquid bio-oil which is a complex mixture of oxygenated hydrocarbons, phenols, and other organic compounds and gaseous products (syngas) which Consists of CO, H_2_, CH_4_, CO_2_, and other gases.

The chemical reactions during biomass pyrolysis are complex and depend on factors such as temperature, heating rate, residence time, and biomass composition [26]. Some of the primary reactions include dehydration (removal of water molecules from the biomass), decarboxylation (removal of carboxyl groups (-COOH) from organic compounds) and dehydrogenation (elimination of hydrogen atoms from organic molecules) [27].

The reaction kinetics of biomass pyrolysis vary with different biomass types and pyrolysis conditions [28]. Higher temperatures and longer residence times generally lead to increased conversion of biomass into biochar, bio-oil, and syngas. The percentage conversion of biomass in pyrolysis processes depends on the specific technology used, operating parameters, and the desired products. Advanced pyrolysis technologies, such as fast pyrolysis and microwave-assisted pyrolysis, can achieve high conversion efficiencies and tailor the product distribution towards biochar, bio-oil, or syngas based on application requirements [25].

### 2.4 Anaerobic digestion

Anaerobic digestion is a biological process that converts organic matter, such as biomass and waste materials, into biogas and digestate in the absence of oxygen. This process occurs naturally in anaerobic environments and is facilitated by a consortium of microorganisms, primarily bacteria and archaea [29]. The chemical reactions involved in anaerobic digestion can be summarized as follows:

i. *Hydrolysis.* Complex organic molecules in the biomass are broken down into simpler compounds such as sugars, amino acids, and fatty acids by hydrolytic enzymes produced by hydrolytic bacteria [30].

Complexorganiccompounds→Sugars,Aminoacids,Fattyacids

ii. *Acidogenesis.* The products of hydrolysis undergo fermentation by acidogenic bacteria, leading to the production of organic acids, alcohols, and volatile fatty acids [31].

Sugars,Aminoacids,Fattyacids→Organicacids,Alcohols,Volatilefattyacids

iii. *Acetogenesis.* Acetic acid and hydrogen are produced from the fermentation products by acetogenic bacteria [32].

Organicacids,Alcohols,Volatilefattyacids→Aceticacid,Hydrogen

iv. *Methanogenesis.* Methanogenic archaea convert acetic acid, hydrogen, and other organic compounds into CH_4_ and CO_2_ through methanogenesis [33].

Aceticacid,Hydrogen,Organiccompounds→Methane,Carbondioxide

Equation (6) represents the overall reaction for anaerobic digestion process.

(6) $$Organic\;matter\to Biogas\left({CH}_{4}+{CO}_{2}\right)+Digestate$$

The percentage conversion of biomass in anaerobic digestion depends on several factors, including the composition of the biomass feedstock, temperature, pH, retention time, and microbial activity. High-quality feedstocks rich in organic matter, such as food waste and animal manure, can achieve higher conversion efficiencies.

### 2.5 Fermentation

Fermentation of biomass is a biochemical process that converts sugars and other organic compounds in biomass into various products, such as ethanol, organic acids, and gases, through the action of microorganisms such as yeasts and bacteria. This process is widely used in the production of biofuels, biochemicals, and other bioproducts from renewable feedstocks. The chemical reactions involved in fermentation depend on the specific type of microorganism and the desired end product. However, the general steps of fermentation can be summarized as follows:

i. *Glycolysis.* Sugars (e.g., glucose) in the biomass are broken down into pyruvate molecules through a series of enzymatic reactions known as glycolysis [34].

(7) Glucose→Pyruvate+ATP

ii. *Fermentation Pathways.* Depending on the microorganism and conditions, pyruvate can undergo different fermentation pathways to produce various end products [35]. During ethanol fermentation, pyruvate is converted into ethanol and carbon dioxide by yeast during alcoholic fermentation.

(8) $$Pyruvate\to Ethanol+{CO}_{2}$$

During lactic acid fermentation, pyruvate is converted into lactic acid by lactic acid bacteria.

(9) Pyruvate→Lacticacid

During acetic acid fermentation, pyruvate is converted into acetic acid by acetogenic bacteria.

(10) Pyruvate→Aceticacid

During butanol fermentation, pyruvate is converted into butanol by certain bacteria and yeasts.

(11) $$Pyruvate\to Butanol+{CO}_{2}$$

The percentage conversion of biomass in fermentation processes depends on factors such as the type of biomass feedstock, fermentation conditions (e.g., temperature, pH, nutrient availability), microbial strains used, and fermentation duration. Different fermentation pathways can lead to varying yields of the desired end products.

## 3 Types of biorefineries

### 3.1 Lignocellulosic biorefinery

Lignocellulosic biomass is considered a second-generation feedstock that provides an alternative to first-generation feedstocks which utilize food/edible crops. As such, it does not directly compete with land requirements for food production [36]. Lignocellulosic biomass has a broad spectrum of plant sources and high availability across tropical and subtropical climates. Global annual generation is estimated at 1.3 billion tons, though currently only 3 % is utilized for bioenergy/bioproducts due to technological challenges [37].

**Fig. 2:**
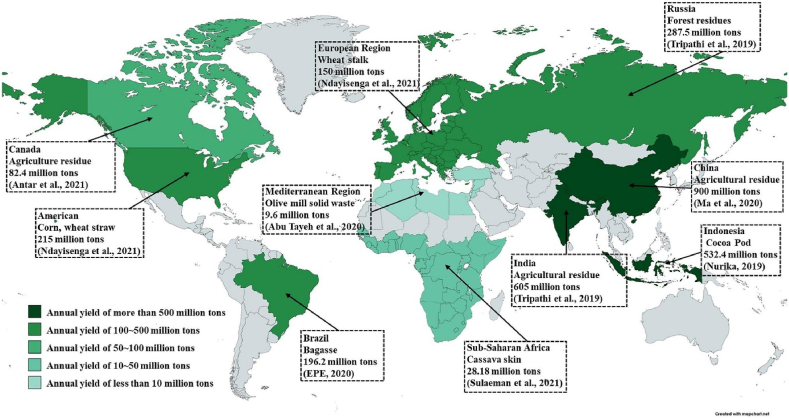
Major lignocellulosic biomass feedstocks and their estimated global generation volumes. Reprinted from Ref. [38], with permission from Elsevier.

**Fig. 3:**
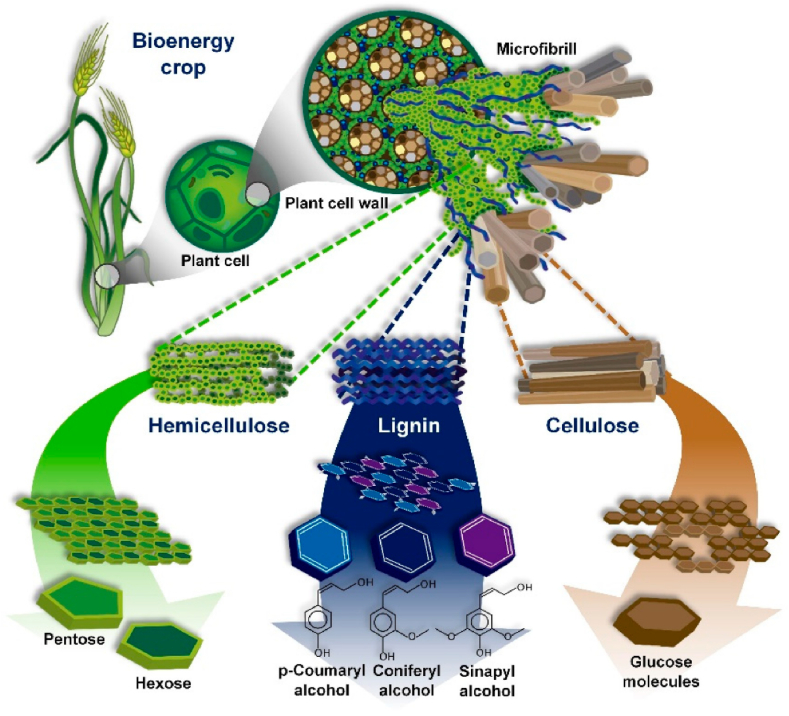
Simplified representation of the chemical structures of the major components that make up lignocellulosic biomass [40].

Major lignocellulosic feedstocks include agricultural residues like barley straw, corn stover, rice straw, and sugarcane bagasse. Other sources are coconut husk, empty fruit bunches, sorghum stalks, woody biomass and municipal solid wastes rich in cellulose. Fig. 2 shows the top lignocellulosic biomass sources and their estimated global volumes. Lignocellulose consists of the polymeric constituents cellulose, hemicellulose and lignin tightly bound together [38]. Fig. 3 presents the chemical structures of these components that makeup plant cell walls.

The lignocellulosic biorefinery concept offers a means of sustainably producing multiple high-value products from this abundant resource. It involves fractionating lignocellulose into its polymeric components through pretreatment and separating steps [38]. Key technologies utilized include thermochemical and biochemical conversions (Fig. 1). De Bhowmick et al. [6] proposed lignocellulosic biorefining as a platform to concurrently develop renewable biofuels and bioproducts through process integration and biomass resource cyclical use.

However, scaling up lignocellulosic conversion technologies and establishing integrated commercial operations faces multifaceted barriers. These include technical challenges, high capital costs, uncertain economics, regulatory and policy gaps as well as limited availability of standardized feedstock supply chains [39]. Addressing such obstacles across the innovation chain is indispensable for large-scale deployment of this biorefinery strategy.

### 3.2 Algal biorefinery

**Fig. 4:**
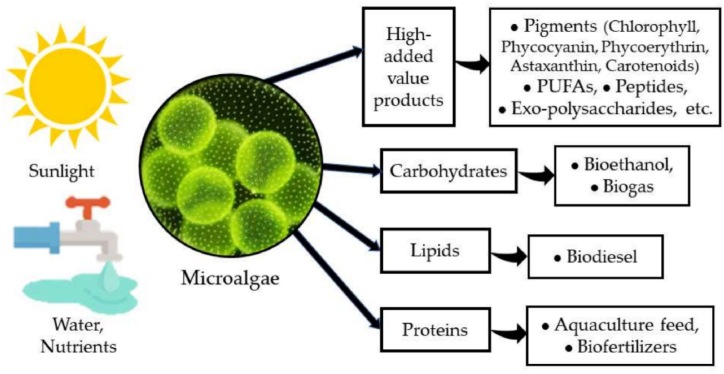
Potential products from microalgae biomass [50].

Algal biomass is considered as advanced (third-generation) biomass feedstocks which provide benefits such as lower land requirement as well as higher biomass productivity and yield when compared with lignocellulosic biomass. Algal biomass can be categorized into two main categories such as macroalgae and microalgae biomass [41]. Fig. 4 shows the various high-value products that can be derived from microalgae biomass through downstream processing. Microalgae cultured photoautotrophically or heterotrophically accumulate lipids, carbohydrates, proteins and pigments. Lipids can be extracted and converted to biodiesel fuel through transesterification. Carbohydrates undergo fermentation to produce biogas and bioethanol. Proteins find application as aquaculture feed and biofertilizers. Additionally, high-value co-products like pigments, antioxidants and pharmaceuticals can be obtained from dedicated algae strains. By harnessing the diverse chemical composition of microalgae, an integrated “algal biorefinery” approach maximizes carbon capture and nutrient recycling from aquatic photosynthesis into multiple end-use commodities.

Microalgae are photosynthetic microorganisms that efficiently utilize solar energy to amass biomass which consists of essential biological compounds [41]. Microalgae can be grown in various reactor systems including photobioreactors which can be vertically designed and fabricated offering lesser land requirements than conventional means of cultivation [42]. Various biorefinery pathways, especially for microalgae biomass, have been proposed to sustainably produce numerous microalgal-based products [43].

Macroalgae are marine microorganisms known as seaweeds which are primarily grown offshore and are abundant on coastal shorelines [44]. They offer a sustainable source of bio-compounds that can be converted to food and high-value products such as biofuels and biochemicals. The development of macroalgae-based biorefinery was introduced for the production of high-valued bio-products from seaweeds [45,46].

Microbes are microorganisms that live as a single-cell or thrive in a multi-celled environment wherein it facilitates the breakdown of hemicelluloses and cellulose from lignocellulosic biomass into bio-compounds essential for fermentation [47]. Through fermentation, biofuels can be generated using the microbial biorefinery systems [48]. Ashokkumar et al. [49] performed a critical review of various microbes used for biofuel production from lignocellulosic biomass in a biorefinery perspective.

### 3.3 Waste biorefinery

Biorefining of wastes provides a viable option for producing bioenergy and bioproducts in a more sustainable manner. Wastes include non-edible biomass and various organic residues generated across multiple economic sectors. Given their heterogeneous and decentralized nature, waste streams present both challenges and opportunities for reuse in a circular bioeconomy framework [51].

**Fig. 5:**
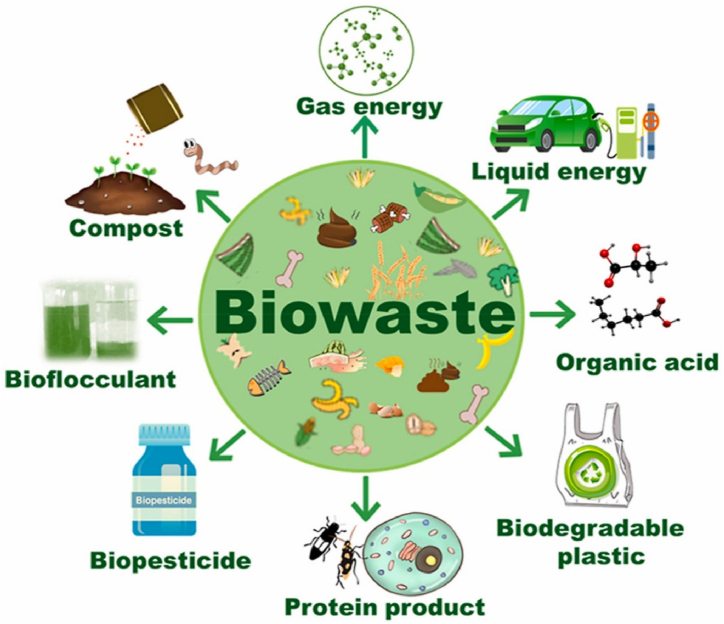
Platform chemicals and high-value products from biowaste generated in a biorefinery. Reprinted from Ref. [58], with permission from Elsevier.

Key wastes that have been explored in biorefining include municipal solid waste [52], food wastes [51], lignocellulosic residues from agriculture/forestry [22], paper/pulp industry sludge [53] and livestock manure [54]. Comprehensive waste characterization is important to identify suitable conversion routes. Fig. 5 illustrates the various energy and material outputs that can be derived from biowaste feeds in an integrated biorefinery. Through thermochemical and biochemical conversion processes, wastes have the potential to produce:

• Energy in the form of biogas via anaerobic digestion, or syngas from gasification, that can be used for heat and power.
• Organic acids such as lactic acid, citric acid and succinic acid via fermentation, for use in pharmaceuticals, food and plastics production.
• Biodegradable bioplastics like polyhydroxyalkanoates that can replace fossil-based plastics.
• Protein products for application in animal feed supplements through conversion of food waste components.
• Biopesticides created by growing beneficial microbes on agro-industrial wastes as substrate.
• Flocculants for water treatment extracted from algal or fungal biomass grown on waste nutrients.
• Compost as a value-added soil amendment from the decomposed solid fraction after energy and chemicals extraction.

Studies have optimized biorefining systems for various waste streams. Mountraki et al. [55] proposed and demonstrated a generic optimization framework to model the integrated treatment of wastewater and waste using a graph-based systems approach, with application to a lignocellulosic biorefinery case. Esteban et al. [56] proposed a food waste biorefinery for chemicals and fuels. Integration of social, economic and environmental factors is important for sustainability [57].

However, operationalizing large-scale, regional waste biorefineries faces challenges around collection logistics, inconsistent input quality, and high capital costs. Concerted efforts are still needed to scale technologies and incentivize private sector investment**.**

## 4 Circular economy and biorefineries

**Fig. 6:**
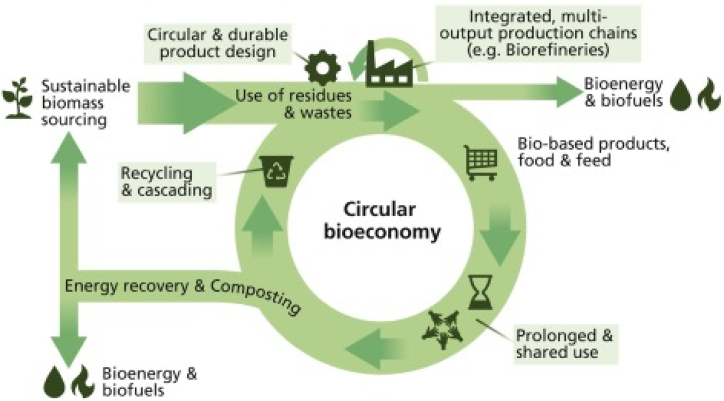
Illustration of the circular economy framework in biorefinery operations [59].

Biorefineries are integral components of the circular economy paradigm, embodying principles of resource efficiency, waste valorization, and sustainable material cycles. Within this framework, biorefineries contribute significantly to minimizing waste and maximizing resource use by converting diverse biomass feedstocks such as agricultural residues, forestry waste, and organic byproducts into valuable biofuels, biochemicals, and bioproducts [4]. This process not only transforms waste materials into high-value outputs but also reduces reliance on virgin resources, promoting a circular approach to resource utilization. Biorefineries optimize resource efficiency through the utilization of various conversion technologies, including thermochemical and biochemical processes, which extract multiple products from a single biomass source [17]. This approach maximizes the use of biomass components, minimizes waste generation, and enhances overall resource productivity. Furthermore, biorefineries operate within closed-loop systems by integrating waste recycling and byproduct utilization, ensuring that residues and byproducts generated during biomass processing are recycled back into the system as inputs for secondary processes or converted into additional value-added products. This closed-loop approach not only minimizes environmental impact but also fosters a circular material flow within the biorefinery ecosystem. Moreover, bio-based products produced in biorefineries contribute significantly to carbon sequestration and climate change mitigation by replacing fossil-based alternatives. Biofuels, bioplastics, and biomaterials derived from sustainable biomass sources help reduce greenhouse gas emissions and support a more sustainable carbon cycle [6]. In essence, the symbiotic relationship between biorefinery operations and circular economy principles underscores the pivotal role of biorefineries as key enablers of sustainable and regenerative industrial practices, driving the transition towards a circular economy model. This concept is illustrated in Fig. 6, depicting the circular economy framework within biorefinery operations.

## 5 Obstacles across the innovation chain

### 5.1 Technical obstacles

#### 5.1.1 Feedstock availability and variability

Feedstock availability and variability presents a significant obstacle to the successful implementation and commercialization of biorefineries. The consistent and sustainable supply of biomass feedstock is crucial for the efficient and cost-effective operation of biorefineries [60]. However, the availability and variability of feedstock pose substantial challenges. The quality, quantity, and seasonality of biomass feedstock can greatly affect the feasibility and performance of biorefinery facilities.

One of the main challenges is the limited availability of suitable biomass feedstock. The demand for biomass resources from various sectors, such as agriculture, forestry, and energy, can create competition and strain on the available feedstock supply [61]. Additionally, the geographical distribution and accessibility of biomass resources can vary, leading to regional disparities in feedstock availability [62]. This can result in logistical challenges and increased costs associated with feedstock transportation, which can impact the overall viability of biorefinery projects.

Moreover, the variability of feedstock presents operational challenges for biorefineries. Biomass feedstock can exhibit fluctuations in composition, moisture content, and energy density, which can affect process efficiency and product quality [62]. The inconsistent feedstock characteristics require additional pre-processing steps and adjustments in biorefinery operations, leading to increased complexity and costs.

To address these challenges, strategies such as feedstock diversification, improved agronomic practices, and advanced harvesting and logistics systems are being explored [63]. Sustainable biomass production systems, including energy crops and agricultural residues, can help mitigate feedstock availability concerns [64]. Furthermore, collaborations between biomass producers, biorefinery operators, and policymakers are crucial to establishing robust supply chains and ensuring a continuous and reliable feedstock feed to biorefineries [65].

#### 5.1.2 Technological maturity

Technological maturity represents a significant obstacle to the successful implementation and commercialization of biorefineries. While biorefinery technologies hold great promise for converting biomass into valuable products, many of these technologies are still in the early stages of development and lack the necessary maturity for large-scale implementation [66].

One of the primary challenges is the need for further technological advancements and optimization. Biorefineries employ complex processes involving biomass pretreatment, enzymatic hydrolysis, fermentation, and product separation. Each step requires continuous improvement and fine-tuning to enhance efficiency, yield, and overall process economics [67]. Scaling up these processes from the laboratory to commercial scale can introduce new technical challenges and operational complexities that need to be addressed.

Another aspect of technological immaturity is the limited integration of multiple conversion pathways. Biorefineries aim to produce a diverse range of products such as biofuels, biochemicals, and biomaterials. However, the integration of various conversion pathways within a single biorefinery is a complex task that requires sophisticated process design and optimization. The lack of mature and integrated technologies for simultaneous production of multiple products poses challenges in terms of process efficiency, product quality, and economic viability [63].

Furthermore, the lack of standardized protocols and benchmarking criteria for biorefinery technologies hampers the assessment of their readiness for commercial deployment. The absence of consistent evaluation metrics and performance indicators makes it difficult to compare and select the most suitable technologies for specific applications [68]. This can lead to uncertainty and reluctance among investors and potential stakeholders, hindering the commercialization of biorefineries.

To overcome these technological challenges, increased research and development efforts are required to advance the maturity of biorefinery technologies. Collaborative initiatives between industry, academia, and government institutions are necessary to foster innovation and accelerate the development of robust, scalable, and economically viable biorefinery platforms [69]. Additionally, greater emphasis on pilot-scale and demonstration projects can help bridge the gap between laboratory-scale proof-of-concept and commercial implementation, enabling the validation and optimization of emerging technologies [70].

#### 5.1.3 Product diversification

Product diversification presents a significant obstacle to the successful implementation and commercialization of biorefineries. While the concept of biorefineries revolves around the production of multiple value-added products from biomass, the practical challenges associated with product diversification can hinder their efficient operation [60].

One of the primary challenges is the complexity of process design and optimization. Biorefineries often aim to produce a range of products such as biofuels, biochemicals, and biomaterials, each with distinct specifications and market demands. However, the simultaneous production of multiple products requires intricate process configurations, integration of various conversion pathways, and careful management of intermediate streams [71]. The design and optimization of such complex processes add technical complexities and increase the risk of operational inefficiencies.

Moreover, product diversification can lead to conflicts in terms of resource allocation and process economics. Different products may have varying market demand, pricing, and profitability. This creates challenges in determining the optimal allocation of biomass feedstock and process streams to maximize the overall economic viability of the biorefinery [60]. Additionally, the production of certain high-value products may require specialized equipment or additional processing steps, which can further increase capital and operational costs.

Furthermore, the market uncertainties and regulatory frameworks surrounding the different product sectors pose challenges for biorefinery operators. The demand and market dynamics for biofuels, biochemicals, and biomaterials can vary significantly, influenced by factors such as policy support, market competition, and consumer preferences [72]. The unpredictable nature of these markets can affect the profitability and long-term sustainability of biorefinery ventures, making it challenging for operators to make informed investment decisions.

To address these challenges, a comprehensive approach is required. Integrated process modeling and optimization techniques can help identify the optimal product portfolio and process configurations that maximize the overall profitability and resource utilization [73]. Market analysis and strategic planning are essential to align the product diversification strategies with market opportunities and identify niche markets with potential high-value products [74]. Additionally, fostering collaborations between biorefinery operators, industry stakeholders, and policymakers can help create a supportive framework for product diversification and market development [75].

### 5.2 Economic obstacles

#### 5.2.1 Capital intensity

Capital intensity represents a significant obstacle to the successful implementation and commercialization of biorefineries. The establishment and operation of biorefineries require substantial investments in infrastructure, equipment, and technology, making them highly capital-intensive ventures [76].

One of the primary challenges is the high upfront capital costs associated with biorefinery construction. The scale and complexity of biorefinery facilities, which typically involve multiple processing units and integrated systems, contribute to significant capital requirements [77]. The need for specialized equipment, such as biomass pretreatment units and fermentation tanks, further adds to the capital costs. These high initial investments pose financial risks and can deter potential investors and project developers.

Moreover, the long payback period of biorefinery projects presents a challenge in attracting financing and securing adequate returns on investments. Biorefineries often require several years to reach full production capacity and generate revenues, primarily due to the time-consuming process of technology development, scale-up, and optimization [60]. The extended payback period increases the financial risks associated with biorefinery investments, as the return on investment may be uncertain or delayed.

In addition, the uncertainty surrounding the market demand and pricing for biorefinery products can further exacerbate the capital intensity challenge. The market dynamics for biofuels, biochemicals, and biomaterials are influenced by various factors, including policy support, competition, and consumer preferences [72]. The volatility and unpredictability of these markets can impact the profitability and long-term financial sustainability of biorefinery projects, making it challenging to attract the necessary capital investments.

To address the capital intensity challenge, various strategies can be employed. Collaborative efforts between industry, government, and financial institutions can help establish supportive financing mechanisms, such as subsidies, tax incentives, and loan guarantees, to alleviate the financial burden on biorefinery projects [78]. Additionally, innovative financing models, such as public-private partnerships and strategic alliances, can facilitate access to capital and mitigate the risks associated with biorefinery investments [79].

Furthermore, continuous efforts to improve the cost-effectiveness and efficiency of biorefinery technologies are essential. Technological advancements, process optimization, and economies of scale can help reduce the capital intensity of biorefineries by enhancing production yields, lowering operational costs, and improving overall process economics [60]. Research and development initiatives focused on cost reduction and process simplification are crucial for making biorefineries more financially viable.

#### 5.2.2 Cost competitiveness

Cost competitiveness represents a significant obstacle to the successful implementation and commercialization of biorefineries. While the production of biofuels, biochemicals, and biomaterials from biomass has the potential to offer sustainable alternatives to fossil-based products, the cost of biorefinery operations often hinders their economic viability [80].

One of the primary challenges is the high cost of feedstock procurement and logistics. Biomass feedstock, such as agricultural residues, dedicated energy crops, or forestry residues, can be expensive to acquire, transport, and store [81]. The availability and cost of feedstock vary geographically, and establishing a reliable and cost-effective supply chain poses challenges for biorefineries. Additionally, competition for biomass resources from other sectors, such as the food and feed industries, can further drive up the cost of feedstock [82].

Furthermore, the cost of conversion technologies and processes remains a significant barrier. Many of the advanced conversion technologies used in biorefineries, such as enzymatic hydrolysis, fermentation, and thermochemical processes, are still in the early stages of development and deployment [60]. These technologies often require specialized equipment, catalysts, enzymes, and other inputs that can be costly. The scale-up and commercialization of these technologies are essential to achieve cost competitiveness, but the associated research, development, and infrastructure investments can be substantial.

Moreover, the economies of scale play a crucial role in the cost competitiveness of biorefineries. Small-scale biorefineries often face challenges in achieving economies of scale, resulting in higher production costs compared to larger facilities [83]. The optimal scale of biorefineries depends on various factors, including feedstock availability, market demand, and technological maturity. However, the capital investment required to build larger-scale biorefineries can be significant, posing financial challenges for project developers.

To overcome the cost competitiveness challenge, various strategies can be employed. Technological advancements and process optimization are essential for reducing the cost of conversion technologies and improving overall process efficiencies [60]. Research and development efforts should focus on cost reduction, yield improvement, and process simplification to enhance the economic feasibility of biorefineries. Additionally, government policies and incentives, such as tax credits, grants, and subsidies, can help offset the capital and operational costs associated with biorefinery investments [84]. Collaborative efforts between industry stakeholders, academia, and government agencies are crucial in fostering innovation, knowledge sharing, and cost reduction throughout the biorefinery value chain [75].

#### 5.2.3 Market demand and price volatility

Market demand and price volatility pose significant obstacles to the successful implementation and commercialization of biorefineries. The market dynamics for biofuels, biochemicals, and biomaterials are influenced by various factors, including policy support, competition, and consumer preferences [75].

One of the challenges is the uncertainty surrounding market demand for biorefinery products. The demand for biofuels, biochemicals, and biomaterials is influenced by factors such as government mandates, environmental regulations, and consumer preferences [85]. Changes in policies or shifts in consumer behavior can significantly impact market demand, making it challenging to predict and plan for biorefinery production. This uncertainty can discourage potential investors and project developers, as it poses risks to the long-term financial sustainability of biorefinery ventures.

Moreover, price volatility in the bioenergy market adds to the complexity of implementing biorefineries. The prices of biomass feedstock, energy commodities, and competing fossil-based products can fluctuate significantly [72]. These price fluctuations can impact the profitability and competitiveness of biorefinery products, making it challenging to establish stable pricing structures and secure consistent revenues. Price volatility also affects the cost-effectiveness of biorefinery operations, as it can disrupt supply chains, alter input costs, and impact the overall economics of the production process.

To address the challenges associated with market demand and price volatility, several strategies can be employed. Diversification of product portfolios can help mitigate risks by reducing reliance on a single product or market [86]. Biorefineries can explore opportunities to produce a range of bio-based products, including biofuels, high-value chemicals, and biomaterials, to cater to different market segments and increase revenue streams. This diversification can help biorefineries adapt to changing market demand and mitigate the impact of price volatility.

Additionally, fostering stable policy frameworks and long-term commitments from governments can provide a more favorable investment climate for biorefineries. Clear and consistent policies, such as renewable energy targets, carbon pricing mechanisms, and tax incentives, can provide stability and predictability for biorefinery developers [87]. These policy measures can create a supportive market environment that encourages investment and reduces the risks associated with market demand and price fluctuations.

Furthermore, collaboration among industry stakeholders, research institutions, and policymakers is crucial for market development and creating a robust demand for biorefinery products. Market development initiatives can focus on building awareness, educating consumers, and fostering partnerships with end-users and industries that can benefit from bio-based products [75]. By establishing strong market linkages and value chains, biorefineries can enhance market demand and create a more stable customer base.

### 5.3 Regulatory obstacles

#### 5.3.1 Policy and regulatory frameworks

Policy and regulatory frameworks can present notable obstacles to the successful implementation and commercialization of biorefineries. While government support and regulations play a crucial role in promoting the development of bio-based industries, the complexity and inconsistency of policies can hinder the growth and investment in biorefinery projects [66].

One of the challenges is the lack of long-term policy stability. Biorefineries require significant capital investments and have long project lifecycles. However, the absence of consistent and predictable policy frameworks can create uncertainty for investors and project developers [75]. Changes in government policies, such as subsidies, tax incentives, or renewable energy mandates, can significantly impact the financial feasibility of biorefinery projects. The absence of stable policies makes it difficult for investors to assess risks and returns accurately, which can deter the necessary investments in the sector.

Moreover, regulatory frameworks can be complex and fragmented, leading to additional barriers for biorefinery implementation. Permitting and compliance processes can be time-consuming and costly, requiring adherence to multiple regulations from various government agencies [87]. The lack of streamlined and harmonized regulations specific to biorefineries can result in delays, increased costs, and inefficient project development. Additionally, regulatory uncertainties related to environmental, health, and safety standards can further impede the progress of biorefineries.

Furthermore, conflicting policies and objectives across different government departments or levels of governance can create challenges. Biorefineries often operate at the intersection of energy, agriculture, and environmental sectors, which are subject to different policies and regulations [75]. The lack of coordination among these sectors can lead to conflicting priorities or overlapping regulations, making it difficult for biorefinery projects to navigate the regulatory landscape effectively.

To overcome these obstacles, it is crucial to establish clear, stable, and supportive policy frameworks. Long-term policy commitments that provide stability and predictability can incentivize investment in biorefineries [88]. Governments can consider implementing renewable energy targets, feed-in tariffs, tax incentives, and other mechanisms that encourage the production and consumption of bio-based products. Additionally, streamlining and harmonizing regulations specific to biorefineries can reduce administrative burdens and provide clarity for project developers [75].

Furthermore, fostering cross-sectoral collaboration and coordination among government agencies is essential. Integrated approaches that bring together energy, agriculture, and environmental sectors can facilitate the development of comprehensive and coherent policies and regulations for biorefineries. This collaboration can help align objectives, address conflicts, and streamline regulatory processes.

#### 5.3.2 Permitting and compliance

Permitting and compliance processes present significant obstacles to the successful implementation and commercialization of biorefineries. While regulations are essential to ensure environmental protection and public safety, the complex and time-consuming nature of permitting requirements can delay and hinder biorefinery projects [89].

One of the challenges is the lengthy and uncertain permitting process. Biorefineries often require multiple permits from various regulatory agencies at different levels of government. These permits may involve environmental impact assessments, air quality permits, water discharge permits, and waste management permits, among others [89]. Coordinating and obtaining these permits can be time-consuming and costly, leading to project delays and increased expenses. The uncertainty associated with the permitting process adds further complexity, as the timeline for obtaining permits can be unpredictable, making it challenging for biorefinery developers to plan and execute their projects efficiently.

Moreover, compliance with stringent environmental regulations can pose challenges for biorefineries. The production processes and waste streams associated with biorefineries may require adherence to strict emission limits, effluent standards, and disposal regulations. Meeting these requirements may involve the installation of pollution control technologies, wastewater treatment systems, and waste management infrastructure [90]. The high costs associated with compliance can be a significant burden, particularly for smaller or emerging biorefinery projects that may struggle to allocate the necessary resources.

Additionally, navigating the regulatory landscape and understanding the applicable regulations can be complex for biorefinery developers. The regulatory frameworks for biorefineries often involve overlapping or conflicting regulations from different agencies or jurisdictions. Keeping up with the evolving regulatory landscape and ensuring compliance can be challenging, particularly for new entrants in the biorefinery sector [91]. The lack of clear guidance and standardized procedures can further complicate the compliance process.

To address these obstacles, it is crucial to streamline and simplify the permitting and compliance processes for biorefineries. Governments can consider developing clear and standardized guidelines specific to biorefinery projects. These guidelines can provide clarity on the required permits, procedures, and timelines, facilitating a more efficient and predictable permitting process [92]. Additionally, regulatory agencies can work towards harmonizing regulations and coordinating their efforts to reduce duplication and streamline compliance requirements.

Furthermore, providing support and resources to assist biorefinery developers in navigating the regulatory landscape can be beneficial. This can include establishing dedicated regulatory assistance programs, providing access to technical expertise, and offering guidance on compliance strategies. Such support can help alleviate the compliance burden and ensure that biorefinery projects can meet the necessary regulatory requirements effectively.

### 5.4 Social obstacles

#### 5.4.1 Public perception and acceptance

Public perception and acceptance can present significant obstacles to the successful implementation and commercialization of biorefineries. The perception and attitudes of the public towards biorefinery projects, particularly in relation to potential environmental impacts and social concerns, can shape the level of support and acceptance from local communities [93].

One of the challenges is the lack of awareness and understanding of biorefinery technologies and their potential benefits. Biorefineries offer the opportunity to produce biofuels, biochemicals, and biomaterials from renewable biomass, contributing to the reduction of greenhouse gas emissions and the transition to a more sustainable economy [66]. However, the general public may have limited knowledge about biorefineries and may hold misconceptions or concerns about their operations and potential environmental impacts. This lack of awareness and understanding can lead to skepticism, resistance, and opposition to biorefinery projects.

Moreover, concerns related to environmental impacts, such as land use change, water consumption, and air emissions, can influence public acceptance of biorefineries. While biorefineries aim to utilize sustainable biomass feedstocks, there can be concerns about the potential competition with food crops, impacts on biodiversity, and the overall sustainability of biomass sourcing [9]. The perception of these potential environmental impacts can lead to public opposition and hinder the development and operation of biorefineries.

Additionally, social and community-related concerns can affect public acceptance of biorefinery projects. Some communities may have concerns about the potential impacts on local economies, employment, and quality of life. The fear of negative social and economic consequences, such as job displacement or changes in land use patterns, can generate resistance and opposition to biorefineries [94]. Building trust and addressing these social concerns are crucial for gaining public acceptance and support.

To overcome these obstacles, effective communication and public engagement strategies are essential. Biorefinery developers should engage in proactive and transparent communication with the public to educate and inform about the potential benefits and impacts of biorefineries [87]. This can involve public consultations, community meetings, and the dissemination of accurate and accessible information. Engaging stakeholders, including local communities, environmental groups, and policymakers, can help address concerns, address misconceptions, and build trust.

Furthermore, incorporating sustainability principles into biorefinery design and operation can enhance public acceptance. Demonstrating the adoption of best practices, such as sustainable sourcing of biomass, efficient resource use, and robust environmental management, can help alleviate concerns about environmental impacts [95]. Engaging with local communities to understand their specific needs and interests and incorporating their input into project design and planning can also foster a sense of ownership and increase acceptance.

#### 5.4.2 Stakeholder engagement

Stakeholder engagement can pose significant obstacles to the successful implementation and commercialization of biorefineries. Effective engagement with various stakeholders, including local communities, environmental organizations, regulatory agencies, and industry partners, is essential for building trust, addressing concerns, and garnering support for biorefinery projects [96].

One of the challenges is the diverse range of stakeholder interests and perspectives. Different stakeholders may have conflicting priorities, expectations, and concerns regarding biorefinery projects. For example, local communities may be concerned about potential environmental impacts and changes to their quality of life, while industry partners may focus on economic viability and market opportunities [97]. Balancing these diverse interests and finding common ground can be complex and time-consuming, requiring effective communication and negotiation skills.

Moreover, stakeholder engagement can be hindered by power imbalances and lack of inclusivity. Certain stakeholders, such as large corporations or government agencies, may have more resources and influence, leading to unequal power dynamics in decision-making processes. The exclusion of marginalized or underrepresented groups from the engagement process can undermine the legitimacy and credibility of biorefinery projects [98]. Ensuring meaningful participation and inclusivity of all stakeholders is critical for fostering trust and achieving successful project implementation.

Additionally, the timing and extent of stakeholder engagement can affect project outcomes. Early and continuous engagement with stakeholders is important to identify concerns, gather input, and address potential issues from the early stages of project development. However, insufficient or delayed engagement can lead to misunderstandings, conflicts, and delays in project implementation [99]. Engaging stakeholders at various stages of the project and providing opportunities for meaningful input and dialogue can help mitigate potential obstacles.

To overcome these obstacles, a proactive and inclusive stakeholder engagement approach is crucial. Biorefinery developers should prioritize early engagement with stakeholders and involve them in decision-making processes [99]. This can include public consultations, stakeholder workshops, and the establishment of advisory groups or committees. Transparent and accessible communication channels should be established to disseminate project information, address concerns, and provide updates on project progress. Incorporating stakeholder feedback and incorporating it into project design and planning can enhance the social acceptance and viability of biorefinery projects.

Furthermore, building long-term relationships with stakeholders can foster trust and collaboration. Regular communication, ongoing dialogue, and responsiveness to stakeholder concerns are essential for maintaining positive relationships [98]. Engaging stakeholders beyond regulatory requirements and involving them in monitoring and evaluation processes can demonstrate commitment to transparency and accountability.

## 6 Recommendations and future outlook

### 6.1 Technological advancements

Significant advancements across core biorefinery technologies are needed to address key technical challenges and enable successful commercialization. Priority research areas should include developing higher-yielding and more robust biomass conversion processes, improving integration capabilities for simultaneous production of multiple products, and scaling up technologies to industrial levels. Collaborative R&D between industry, academia and government is critical to accelerate technology development through shared resources and expertise. Pilot and demonstration projects play a vital role in validating emerging technologies at commercial-scale and identifying areas for further optimization. Continuous improvements in yields, selectivity, efficiency and cost-competitiveness will be important to make biorefineries economically viable.

### 6.2 Process optimization and design

Advanced process modeling, simulation and integration techniques should be leveraged to optimize biorefinery configurations for maximum resource and energy efficiency. Areas of focus include optimal product portfolios based on synergies between conversion pathways, optimal allocation of feedstock and process streams, integrated waste and byproduct utilization. Life cycle assessment of biorefinery systems can help identify areas for reducing environmental impacts. Modular, scalable and flexible biorefinery designs will allow operators to adapt to changing market dynamics and feedstock availability. Process intensification approaches may also help address challenges related to capital intensity.

### 6.3 Supportive policy frameworks

Long-term national bioeconomy strategies and implementation roadmaps are crucial for establishing policy certainty. Policy tools such as renewable energy targets, low carbon fuel standards, carbon pricing, tax incentives and public procurement commitments can drive demand for bio-based products and investments in biorefinery capacity. Strategies are also required for sustainable biomass production and supply chains. Regulatory frameworks need to provide clarity while streamlining permitting requirements. Cross-sectoral collaboration between energy, agriculture and environmental agencies is important for developing coherent policies. Monitoring progress and adjusting policies based on lessons learned will be important.

### 6.4 Financing mechanisms

Innovative financing models involving public-private partnerships and collaboration across finance, industry and government sectors can help mobilize large capital investments. Loan guarantee programs and results-based financing approaches may help address risks. Project incubators and accelerators can support early-stage biorefinery ventures. Blended public-private financing vehicles targeting both equity and debt can draw greater institutional capital. International cooperation on commercializing innovative financing approaches will also be important. Continued R&D support for cost reduction will also help address challenges related to capital intensity over the long-term.

### 6.5 Market development initiatives

Proactive measures are required to create demand for biorefinery products and address market uncertainties. Strategies could include procurement targets for bio-based materials in government contracts, educational programs to raise awareness on bioeconomy benefits, and certification standards for verifying sustainability claims. Fostering strategic partnerships between industry stakeholders across supply chains can help establish robust market linkages. Incentivizing private sector participation in research, demonstration and early adoption can help accelerate commercialization.

### 6.6 Stakeholder engagement

Biorefinery developers must adopt principled and inclusive approaches to stakeholder engagement. This includes early and ongoing consultation with affected communities and groups, addressing diverse concerns, and incorporating feedback into project planning. Engagement strategies need to consider issues of representation, participation, consent and accountability. Regular status updates and transparency on environmental performance can help build trust. Multi-stakeholder advisory bodies composed of local leaders, indigenous peoples, youth, women and underrepresented groups may enhance social license to operate.

## 7 Conclusions

The successful commercial deployment of biorefineries holds tremendous potential to advance the bioeconomy and enable the transition to more sustainable industrial systems. However, numerous obstacles must be overcome across the entire biorefinery innovation chain before this potential can be fully realized. The key findings from this study are:

• Continued advancements in optimizing biomass feedstocks are needed to ensure consistent quality and reliable supply. Effectively integrating conversion processes remains challenging due to their complexity. Further research and development is required to enhance the efficiency of these integrated processes.
• The substantial initial investment required to build biorefineries presents a major economic hurdle. Ensuring the competiveness of biorefineries compared to conventional fossil-based industries is crucial. Market fluctuations and uncertainties hamper long-term financial planning and investment due to the challenges of forecasting returns.
• Complex regulations often result in delays and added costs of compliance, creating significant barriers for biorefinery development. Supportive public policies are critical to help mitigate risks and provide a stable regulatory environment that enables the growth of the biorefinery industry.
• Transparent and inclusive engagement with all stakeholders is essential to gain public acceptance for biorefineries. Strategic communication of the environmental and sustainability benefits of biorefineries can help enhance public perception and garner broader support.

Promising strategies for overcoming the barriers to biorefinery deployment include leveraging international pilots and demonstration facilities to provide valuable data and improve economic viability. Additionally, optimizing biomass supply chains can enhance process efficiency and cost-effectiveness. Cross-agency coordination and streamlined regulations can help reduce barriers while safeguarding social and environmental priorities. Furthermore, multi-stakeholder collaboration and innovative financing models are crucial for accelerating innovation and deployment.

## Funding

This research did not receive any specific grant from funding agencies in the public, commercial, or not-for-profit sectors.

## Declaration of generative AI in scientific writing

During the preparation of this work the authors used Grammarly and Anthropic Claude to verify grammatical correctness, enhance readability, and improve language. After using these tools, the authors reviewed and edited the content as needed and takes full responsibility for the content of the publication.

## Data availability statement

No data was used for the research described in the article.

## CRediT authorship contribution statement

**Denzel Christopher Makepa:** Writing – original draft, Conceptualization. **Chido Hermes Chihobo:** Writing – review & editing, Validation.

## Declaration of competing interest

The authors declare that they have no known competing financial interests or personal relationships that could have appeared to influence the work reported in this paper.
